# Periostitis Following Typhoid Fever

**Published:** 1885-03

**Authors:** 


### Periostitis Following Typhoid Fever.

It is not generally realized that periostitis is sometimes a sequela to typhoid fever, but Dr. John D. Hayward, of Liverpool, believes that it is a much more common occurrence than is usually supposed. During the past few years he has met with several cases which he notes in the *British Medical Journal*, Jan. 3, 1885. In one case the periostitis did not suppurate, and the patient did well; in another, periostitis appeared about several of the long bones; suppuration occurred with external discharge; but, after new attacks of periostitis had ceased to occur, and the old sinuses had healed (which they did readily), the patient sank under acute phthisis, of which, previously to the enteric attack, there had been no evidence. The attacks of typhoid fever in which he has noticed this sequela have been severe ones.

With reference to the etiology, Dr. H. considers that it is possible that this local inflammation is due to the tendency to degenerative changes induced by the exhausted condition of the system after a severe enteric fever, resembling thus some of the commoner sequelæ of this fever. Or there may be a septicæmic origin in some cases, just as some of the instances of parotitis, marasmus, and phthisis after enteric fever, are supposed to arise.

---

Dr. F. H. Bosworth reports in the *Medical Record*, that on applying a two per cent. solution of cocaine to the nasal passages, the venous sinuses below the mucous membrane become, within twenty or thirty seconds, so rigidly contracted as to expel all the blood contained in them, and to cause the membrane to cling closely to the bony structure which then becomes sharply outlined. He has used the drug in hypertrophy of the nasal mucous membrane (*nasal catarrh*), acute *coryza*, and in operations for nasal *polypus*. In each case the venous congestion or turgescence was so thoroughly kept down that all discomfort was removed, and in the case of polypi not only the recognition and removal of the growth became quite easy, but also turned out a bloodless operation.

